# Examining neighborhood and interpersonal norms and social support on fruit and vegetable intake in low-income communities

**DOI:** 10.1186/s12889-018-5356-2

**Published:** 2018-04-05

**Authors:** Akilah Dulin, Patricia M. Risica, Jennifer Mello, Rashid Ahmed, Kate B. Carey, Michelle Cardel, Chanelle J. Howe, Sarah Nadimpalli, Kim M. Gans

**Affiliations:** 1 Behavioral and Social Sciences, Center for Health Equity Research, Brown University School of Public Health, Box G-S121-8, Providence, RI 02912 USA; 2 Center for Health Equity Research, Brown University School of Public Health, Box G-S121-8, Providence, RI 02912 USA; 3 Behavioral and Social Sciences, Center for Alcohol and Addiction Studies, Brown University School of Public Health, Box G-S121-5, Providence, RI 02912 USA; 4 Health Outcomes and Policy, Institute for Child Health Policy, University of Florida, 2197 Mowry Road, 132, PO Box 100177, Gainsville, FL 32610 USA; 5 Epidemiology, Brown University School of Public Health, Box G-S121-2, Providence, RI 02912 USA; 6 Human Development and Family Studies, University of Connecticut, 348 Mansfield Road, Unit 1058, Room 320, Storrs, CT 06269 USA

**Keywords:** Neighborhood, Social environment, Resilience, Behavior, Diet

## Abstract

**Background:**

We examined whether neighborhood-, friend-, and family- norms and social support for consumption and purchase of fruits and vegetables (F&V) were associated with F&V intake among low-income residents in subsidized housing communities. We examined baseline data from a study ancillary to the Live Well/Viva Bien intervention. Participants included 290 residents in four low-income subsidized housing sites who were ≥ 18 years of age, English and/or Spanish speaking, and without medical conditions that prevented consumption of F&V.

**Methods:**

Linear regression models examined associations of norms and social support with F&V intake after adjustments for sociodemographic characteristics.

**Results:**

In the analysis, neighborhood social support for F&V was associated with a 0.31 cup increase in F&V intake (95% CI = 0.05, 0.57). The family norm for eating F&V and family social support for eating F&V were associated with a 0.32 cup (95% CI = 0.13, 0.52) and 0.42 cup (95% CI = 0.19, 0.64) increase in F&V intake, respectively.

**Conclusions:**

To our knowledge, no other studies have examined neighborhood, family, and peer norms and social support simultaneously and in relation to F&V intake. These findings may inform neighborhood interventions and community-level policies to reduce neighborhood disparities in F&V consumption.

## Background

The 2015–2020 Dietary Guidelines for Americans recommend that adults consume between 1 to 2 cups of fruit and 1 to 2 ½ cups of vegetables each day depending on caloric intake [1]. However, fruit and vegetable (F&V) intake among adults in the United States (U.S.) is low [1]. On average, only 18% of adults consume the daily recommended intake of fruits and only 14% consume the daily recommended intake of vegetables [2]. Low F&V intake is further exacerbated by factors related to low neighborhood socioeconomic position (SEP) such as neighborhood-level income, poverty, education, and unemployment. Lower neighborhood-level SEP is associated with lower levels of serum carotenoids [3, 4] and earlier onset of diet-related chronic diseases such as some cancers, obesity, type 2 diabetes, and cardiovascular disease [5–7].

Neighborhood conceptual frameworks like the Community Energy Balance Framework and some study findings suggest that neighborhood-level disparities in F&V intake may partially result from characteristics of the food environment that differ by neighborhood-level SEP [8–10]. For example, lower neighborhood-level SEP food environments may be associated with lower F&V intake due to access (i.e., food deserts and food swamps) [11–14]. These urban food deserts (i.e., census tracts where at least 33% of the population lives more than one mile from a supermarket or large grocery store) [15] and food swamps (i.e., census tracts where the high prevalence of access to energy-dense nutrient poor foods disproportionately outweighs access to healthy foods) [16] often exist concurrently [17]. However, research support for the influence of food environments on diet quality has been mixed, with some research evidence, including longitudinal evidence, suggesting that neighborhood food environments may not be associated with diet quality [18, 19].

Differences in social environments (e.g., perceived norms and social support) within low SEP neighborhoods may explain some of the inconsistent findings in the food environment literature [11, 20]. Aspects of the social environment such as neighborhood norms may influence F&V intake [20, 21]. Perceived neighborhood norms may be descriptive (i.e., beliefs about typical dietary behaviors in the neighborhood) or injunctive (i.e., beliefs about acceptable dietary behaviors in the neighborhood) and may partially explain differences in F&V-related behaviors across neighborhoods. Individuals learn about acceptable diet-related norms through observations and interactions with others and may modify their eating-related behaviors based on these observed habits [22, 23]. As such, neighborhood norms may play a role because individuals residing in the same neighborhood live in close proximity with one another. In turn, individuals may learn socially acceptable norms through social interactions and may be influenced to conform to perceptions of normative behaviors [24–26].

Additional characteristics of the neighborhood social environment such as social support may function as resilience resources that partly mitigate the effects of food desert/food swamp environments on dietary intake in neighborhoods of low SEP. Resilience refers to “positive adaptation in the face of ongoing daily stressors and highly taxing, yet still common events [27].” Resilience frameworks such as the Reserve Capacity Model posit that individuals with low-SEP may overcome the adversities of disadvantaged economic environments if they maintain a reserve of adaptive and social resilience resources (e.g., self-esteem, optimism, social support) [28]. Quantitative studies show that resilience may lead to more positive health behaviors and/or may buffer the effects of adversities on physiological functioning [29]. This may suggest that although living in a low SEP food desert may be a chronic stressor, the availability of resilience resources (e.g., neighbor social support), may help individuals to engage in positive behaviors such as healthy eating. Social support may be an important resilience resource because individuals may rely upon their social networks of neighbors and important others like family and friends, to provide informational, instrumental, and/or emotional support related to dietary behaviors [30, 31].

Initial research evidence suggests that neighborhood norms and social support may be associated with diet quality. Study findings have shown that neighborhood social ties are associated with healthy eating among low-income children [32]. Among adults, neighborhood social capital (i.e., an index of neighborhood social support, civic participation, trust, attachment, and cooperation) is associated with F&V intake [33]. While informative, these findings do not include other norms and social support factors that influence diet. Prior work has shown that family and peer norms and social support influence diet quality. These findings indicate that family, friend- and parent-related dietary norms predict unhealthy snacking [34], intake of sugar sweetened beverages, fast food and F&V consumption, and overeating [35–37]. Additionally, a systematic review of the literature on predictors of F&V consumption among adults indicates that friend and family social support for F&V are strong psychosocial determinants of F&V intake [31]. Also, some study findings have shown that people with higher levels of social support such as social interactions and networks, engage in healthier behaviors, including increased F&V consumption [30, 31, 36]. However, Tamers and colleagues suggest that social support and social networks are associated with both healthy and unhealthy dietary behaviors [37].

The aforementioned findings suggest that further examination of the potential influence of neighborhood norms and social support on F&V intake, while also accounting for peer and family norms and social support, may be warranted. Therefore, the objectives of this study is to examine whether and how neighborhood-, friend, and family- norms and social support for F&V are associated with F&V intake among individuals in low-SEP communities. We hypothesize that after adjustment for sociodemographic characteristics, stronger neighborhood, peer, and family norms and social support for F&V will be associated positively with F&V intake.

## Methods

### Study design

We examined baseline data from an ancillary study conducted with participants in the Live Well/Viva Bien F&V intervention. The ancillary study was designed to collect data on potential neighborhood-level mediators and/or moderators of the intervention’s effectiveness. The Live Well/Viva Bien study was a multilevel intervention that delivered monthly mobile F&V markets at discounted costs and nutrition education materials and programs to residents in low-income subsidized housing complexes in Providence County, Rhode Island.

The Live Well/Viva Bien study used a cluster randomized controlled trial design to assign 14 demographically-matched family (*n* = 5) and elderly/disabled (*n* = 9) housing sites to one of two conditions: control or intervention group. We matched housing sites by number of units, type of site (family or elderly/disabled) and race/ethnicity. Housing sites were randomly assigned to either 1) the intervention group which received discounted F&V markets, nutrition education, and healthy food marketing campaigns or 2) the control group which received stress reduction and physical activity materials and a free six-week membership to the YMCA. We conducted surveys at baseline, six-, and 12-month follow-up periods. More detail about the design and rationale for Live Well/Viva Bien is presented elsewhere [38]. The Live Well/Viva Bien study received approval from the Brown University Institutional Review Board and study participants provided their written informed consent.

### Study recruitment and eligibility

Recruitment of housing sites and residents occurred between 2011 and 2013. In order to be eligible, housing sites needed to have at least 190 housing units and at least 90% of residents needed to be able to speak and/or read English or Spanish. The sites also needed to have a low-turnover rate (i.e., < 20%) and the infrastructure to host the markets. To assist with recruitment of participants, research staff hired Resident Assistants who were residents of the housing communities. Resident Assistants posted flyers, informed residents of recruitment events, and placed door hangers containing study-related contact and event information on the door of each housing unit.

In order to be eligible to participate in the evaluation component of Live Well/Viva Bien, participants needed to be ≥18 years of age, be a full-time resident living in the housing complex, and not have any plans to move within the next year. Participants also had to self-report responsibility for at least 50% of the household food shopping. Eligible participants also could not have any medical conditions that would preclude them from participating in study activities. Also, participants needed to be able to read or understand English or Spanish and have access to a DVD player. For the ancillary study, we analyzed data from four of the housing sites. These sites included 1 family site and 4 elderly/disabled sites.

### Measures

#### Dependent variable

##### F&V intake

Participants self-reported F&V intake using the 18 item National Cancer Institute’s (NCI) Eating at America’s Table All Day Screener [39]. The NCI All Day Screener queried foods consumed over the past month. Participants were asked to think about the F&V they usually ate last month and to report the frequency (from never to 5 or more times per day) and serving size (from less than ½ cup to more than 1 ½ cups) for each F&V. The total consumption of F&V was calculated by summing the products of each food group. The responses to the frequency questions were recoded based on the NCI methods to daily averages by multiplying the frequency of consumption by the portion size. For all analyses, we excluded 100% fruit juice and 100% vegetable juice responses.

#### Independent variables

##### Perceived neighborhood norms and social support for F&V

We created 6 original items to examine neighborhood descriptive and injunctive norms for F&V intake and 3 items to explore social support for F&V (see Table 1).

**Table 1:** Neighborhood Norms and Social Support Original Items Examined in the Exploratory Factor Analysis

1. How important is it to your neighbors to buy more fruits and vegetables?^a^
2. How important is it to your neighbors to eat more fruits and vegetables?^a^
3. How much do your neighbors approve of buying fresh fruits and vegetables?^b^
4. The high cost of fruits and vegetables keep your neighbors from buying them as much as they’d like to.^c^
5. People in your neighborhood eat a lot of fruits and vegetables.^c^
6. Do you think that you eat more fruits and vegetables, about the same amount of fruits and vegetables, or fewer fruits and vegetables than your neighbors?^d^
7. During the past 3 months, how often did your neighbors encourage you to buy fruits and vegetables?^e^
8. During the past 3 months, how often did your neighbors encourage you to eat fruits and vegetables?^e^
9. During the past 3 months, how often did your neighbors encourage you to serve your family more fruits and vegetables?^e^

All scales used a Likert type response option ranging from 1 to 5

^a^Injunctive Norms: Response options ranged from “Not at All Important,” to “Extremely Important”

^b^Injunctive Norm - Response options ranged from “Disapprove a Lot” to “Approve a Lot”

^c^Descriptive Norms: Response options ranged from “Disagree a Lot” to “Agree a Lot”

^d^Descriptive Norm: Response options were “Fewer,” “About the Same,” or “More”

^e^Social Support: Response options ranged from “Never” to “Very Often”

We conducted exploratory factor analysis to identify factors within this nine-item group. We conducted a Principal Component Analysis (PCA) using a varimax rotation with no predetermined number of factors. Using this method, we identified four initial factors. However, we removed four individual items from further analysis because they had different response options from the other questions and they did not load such that cogent factors could be identified. The final exploratory factor analysis identified two factors that included the five remaining items which we used in further analysis. *Perceived injunctive neighborhood norms for F&V (Factor 1):* Participants responded to, “How important is it to your neighbors to buy F&V,” and, “How important is it to your neighbors to eat F&V.” Response options ranged from “Not at all Important” to “Extremely Important.” We calculated the mean score which ranged from 1 to 5, with higher scores indicating stronger neighborhood injunctive norms for F&V. *Perceived neighborhood social support for F&V (Factor 2):* Participants responded to three questions including “How often did your neighbors encourage you to buy F&V…eat F&V… and serve your family more F&V.” Response options ranged from “Never” to “Very Often.” Mean scores ranged from 1 to 5, with higher scores corresponding to more social support for F&V. The Cronbach’s Alpha was 0.89.

##### Perceived descriptive neighborhood norm for F&V

None of the descriptive norm items performed well in the PCA because of the differing response options, so we included one descriptive neighborhood norm as a separate item. Participants responded to one item, “People in your neighborhood eat a lot of fruits and vegetables.” Response options ranged from “Disagree a Lot” to “Agree a Lot.”

##### Perceived descriptive family norm for F&V

Participants rated how strongly they agreed with the statement, “People in your family eat a lot of F&V.” Possible scores ranged from 1 to 5 with higher values indicating a stronger family norm for F&V.

##### Perceived friend and family social support for F&V

Participants responded to three items regarding the number of times during the past 3 months that they received support from friends, family, and household members to buy, eat, and serve more F&V [40]. An additional item asked how often family, friends, and household members purchased F&V for their household. Response options ranged from “Never” to “Very Often.” Possible mean scores ranged from 1 to 5 with higher scores indicating more social support for F&V. The Cronbach’s alpha was 0.86.

### Covariates

Participants self-reported their gender (male or female), age (coded as 18-29, 30-39, 40-49, 50-59, 60-69, and 70 years or older), race (American Indian or Alaska Native, Black, White, Asian, Native Hawaiian or Other Pacific Islander, Mixed, or Other), and ethnicity (Hispanic, yes or no). Participants also reported their primary language(s) spoken (English only, Spanish only, Both with more English than Spanish, Both with equal amounts of English and Spanish, Both with more Spanish than English, or other language), highest level of education completed, employment status, and total annual household income for the previous year.

### Statistical analysis

We described participants’ demographic characteristics using means and standard deviations for continuous variables and frequencies and percentages for categorical variables. We conducted t-tests and chi-squared tests to examine whether there were significant differences between participants with and without missing data, if there were significant differences in F&V intake by socio-demographic characteristic, and whether there were significant differences in norms and social support by socio-demographic characteristic. For the main analysis, we used multivariate linear regression instead of generalized estimating equations because of the limited number of housing sites (*N* = 4) where neighborhood data were collected. We examined three multivariate linear regression models to examine associations between norms and social support on fruit intake, vegetable intake, and combined F&V intake. We adjusted all of these models for housing site, gender, age, race, ethnicity, and education. We present unstandardized beta coefficients. We set the probability criterion at α < 0.05 and we used SAS version 9.4 (SAS Institute Inc., Cary, NC) for all analyses.

## Results

A total of 414 residents in four housing sites completed the baseline ancillary study survey questions. Participants who were missing data on race, ethnicity, education, employment and income were omitted from the analyses, which left an analytic sample of 290 participants. There were no significant differences in gender, language spoken in the home, ethnicity, race, or income status between participants with or without missing data. There were also no significant differences in neighborhood norms, family norm, family and peer social support, fruit, vegetable, or combined F&V intake between those with or without missing data. However, participants with missing data were significantly older, more likely to be unemployed, disabled, or retired, and reported less neighborhood social support for F&V (these data are not shown).

Socio-demographics and mean fruit, vegetable, and combined F&V intake for each sociodemographic characteristic are presented in Table 2. The majority of participants were female (80%). Participants were similarly distributed across age intervals ranging from ages 18-29 years to 60-69 years, with a smaller percentage reporting 70 years of age or older. The majority of participants reported that they were White (71%) and 40% of participants were Hispanic. Slightly more than half of the participants reported speaking “English Only” (55.8%). Regarding SEP-related variables, 5% of participants reported that they were employed full-time and almost one-third of participants reported that they were disabled. Slightly more than one-fourth of participants reported having less than a tenth-grade education and 21% reported completing some post-high school training (e.g., vocational, college). Almost half of the participants (46%) reported annual household incomes of less than $12,000.

**Table 2:** Descriptive Characteristics of the Sample and Fruit, Vegetable, and Fruit and Vegetable (F&V) Combined Intake by Participant Characteristic, Mean (95% Confidence Interval) or *N* (%)

	*N* (%)	Fruit	*P*-value	Vegetables	*P*-value	F&V	*P*-value
Gender			0.41		0.76		0.78
Male	59 (20)	1.21 (0.85, 1.57)		2.02 (1.61, 2.44)		3.23 (2.63, 3.83)	
Female	231 (80)	1.38 (1.20, 1.56)		1.95 (1.74, 2.16)		3.33 (3.03, 3.63)	
Age group			0.68		0.32		0.29
18–29	48 (17)	1.38 (0.98, 1.78)		1.85 (1.39, 2.31)		3.23 (2.57, 3.92)	
30–39	45 (16)	1.09 (0.68, 1.50)		1.87 (1.39, 2.34)		2.96 (2.27, 3.64)	
40–49	43 (15)	1.42 (0.99, 1.84)		2.04 (1.55, 2.52)		3.45 (2.75, 4.15)	
50–59	46 (16)	1.52 (1.12, 1.93)		2.10 (1.63, 2.57)		3.63 (2.95, 4.32)	
60–69	54 (19)	1.22 (0.84, 1.60)		1.58 (1.14, 2.71)		2.80 (2.18, 3.42)	
70–79	33 (11)	1.59 (1.11, 2.07)		2.35 (1.80, 2.91)		3.94 (3.14, 4.74)	
80+	21 (7)	1.21 (0.61, 1.81)		2.36 (1.66, 3.45)		3.57 (2.57, 4.57)	
Hispanic			0.40		0.30		0.22
Yes	117 (40)	1.26 (1.01, 1.51)		1.84 (1.55, 2.14)		3.10 (2.68, 3.53)	
No	173 (60)	1.40 (1.19, 1.61)		2.05 (1.80, 2.29)		3.45 (3.10, 3.80)	
Race			0.58		0.41		0.47
White	206 (71)	1.30 (1.11, 1.49)		2.04 (1.82, 2.26)		3.34 (3.02, 3.66)	
Black	21 (7)	1.78 (1.17, 2.38)		2.03 (1.34, 2.73)		3.81 (2.81, 4.81)	
Asian	2 (1)	0.75 (−1.20, 2.70)		2.76 (0.50, 5.41)		3.51 (0.26, 6.76)	
Native Hawaiian or Other Pacific Islander	2 (1)	1.16 (−0.79, 3.11)		2.63 (0.37, 4.88)		3.79 (0.53, 7.44)	
American Indian or Alaska Native	4 (1)	0.90 (−0.48, 2.28)		1.69 (0.10, 3.29)		2.60 (0.30, 4.89)	
Mixed	33 (11)	1.61 (1.13, 2.09)		1.91 (1.36, 2.47)		3.52 (2.72, 4.32)	
Other	22 (8)	1.11 (0.53, 1.70)		1.21 (0.53, 1.89)		2.33 (1.35, 3.31)	
Language			0.61		0.25		0.16
English only	162 (56)	1.33 (1.12, 1.55)		1.96 (1.71, 2.21)		3.29 (2.93, 3.65)	
Spanish only	20 (7)	0.85 (0.23, 1.46)		1.46 (0.75, 2.17)		2.30 (1.28, 3.33)	
Both, more English than Spanish	28 (10)	1.31 (0.79, 1.83)		1.55 (0.95, 2.15)		2.86 (1.99, 3.72)	
Both, equal amounts	20 (7)	1.49 (0.87, 2.10)		2.12 (1.41, 2.83)		3.61 (2.58, 4.63)	
Both, more Spanish than English	34 (12)	1.52 (1.05, 1.99)		2.33 (1.78, 2.87)		3.85 (3.06, 4.63)	
Other Language	26 (9)	1.49 (0.95, 2.03)		2.27 (1.64, 2.89)		3.75 (2.86, 4.65)	
Employment status			0.61		0.04*		0.15
Full time^a^	15 (5)	1.56 (0.85, 2.28)		1.56 (0.74, 2.37)		3.12 (1.94, 4.31)	
Part time^b^	29 (10)	1.14 (0.63, 1.65)		2.41 (1.82, 3.99)^d^		3.56 (2.70, 4.48)	
Unemployed^c^	59 (20)	1.14 (0.78, 1.50)		1.82 (1.41, 2.23)		2.96 (2.36, 3.55)	
Disabled^d^	90 (31)	1.33 (1.04, 1.62)		1.63 (1.29, 1.96)^b,e,f^		2.96 (2.48, 3.44)	
Retired^e^	65 (22)	1.53 (1.19, 1.87)		2.26 (1.87, 2.65)^d^		3.79 (3.22, 4.35)	
Student/Homemaker^f^	32 (11)	1.45 (0.96, 1.94)		2.39 (1.83, 2.94)^d^		3.84 (3.03, 4.65)	
Education			0.63		0.46		0.32
< First grade-9th grade	77 (27)	1.26 (0.94, 1.57)		1.81 (1.45, 2.18)		3.07 (2.54, 3.59)	
Grades 10-12	151 (52)	1.31 (1.08, 1.53)		1.94 (1.68, 2.20)		3.25 (2.87, 3.62)	
Vocational/Tech/some college	52 (18)	1.56 (1.17, 1.94)		2.27 (1.83, 2.71)		3.83 (3.19, 4.46)	
Bachelor’s Degree/Post-graduate	10 (3)	1.51 (0.64, 2.38)		1.96 (0.95, 2.97)		3.46 (2.01, 4.92)	
Income (yearly)			0.44		0.40		0.39
< $6000	17 (7)	1.11 (0.46, 1.77)		1.93 (1.13, 2.73)		3.04 (1.92, 4.15)	
$,6000-$11,9999	94 (39)	1.43 (1.15, 1.71)		1.77 (1.42, 2.11)		3.19 (2.72, 3.67)	
$12,000-$17,999	74 (30)	1.21 (0.90, 1.53)		2.01 (1.63, 2.39)		3.22 (2.69, 3.76)	
$18,000+	58 (24)	1.55 (1.20, 1.91)		2.24 (1.81, 2.68)		3.79 (3.18, 4.49)	

some columns do not add up to 100% due to rounding error

^a–f^denote significant differences between employment status categories

When fruit, vegetable, and F&V combined intake were examined by sociodemographic characteristics, we found significant differences in vegetable intake across employment status type: part-time employees, retirees, students and homemakers consumed more vegetables than disabled individuals (*p* < 0.05). However, there were no significant differences in vegetable intake between participants who were employed full-time or who were disabled.

Table 3 presents the results testing for significant differences in neighborhood-, friend-, and family- norms and social support by socio-demographic characteristics. Participants who were Hispanic, reported speaking more English than Spanish, and had a twelfth grade education or less than a 10th grade education reported a higher perceived neighborhood descriptive norm for the amount of F&V consumed by neighbors than participants who were not Hispanic, spoke English only, and who reported some post-high school education. The family norm for F&V significantly differed by ethnicity with Hispanic participants reporting a higher family norm for F&V. For family and peer social support for F&V, participants who were Hispanic, spoke equal amounts of Spanish and English, and reported an income of less than $6000, reported more friend and family social support for F&V than those who were not Hispanic, all other language groups, and those with higher incomes.

**Table 3:** Mean and 95% Confidence Interval for the Norm and Social Support Measures Presented by Participant Characteristic

	Neighborhood Injunctive norm for F&V	Neighborhood Descriptive Norm F&V	Neighborhood Social support for F&V	Family Norm for F&V	Family and Peer Social Support for F&V
Gender					
Male	3.04 (2.77, 3.32)	3.37 (3.01, 3.73)	1.80 (1.52, 2.27)	4.05 (3.70, 4.41)	2.80 (2.49, 3.11)
Female	3.25 (3.11, 3.39)	3.31 (3.13, 3.49)	1.70 (1.56, 1.84)	4.05 (3.87, 4.23)	2.50 (2.35, 2.66)
Age group					
18–29	3.13 (2.82, 3.43)	3.35 (2.95, 3.75)	1.79 (1.48, 2.17)	4.42 (4.03, 4.81)	2.81 (2.47, 3.16)
30–39	3.23 (2.92, 3.55)	3.42 (3.01, 3.83)	1.43 (1.12, 1.74)	4.33 (3.93, 4.74)	2.69 (2.33, 3.25)
40–49	3.13 (2.81, 3.45)	3.26 (2.83, 3.68)	1.61 (1.29, 1.93)	4.00 (3.59, 4.41)	2.65 (2.28, 3.62)
50–59	3.58 (3.27, 3.89)	3.37 (2.96, 3.78)	1.75 (1.43, 2.56)	3.85 (3.45, 4.25)	2.39 (2.04, 2.74)
60–69	3.15 (2.86, 3.43)	3.41 (3.03, 3.78)	1.81 (1.52, 2.12)	3.96 (3.60, 4.33)	2.44 (2.11, 2.77)
70–79	3.09 (2.72, 3.46)	3.52 (3.03, 4.35)	1.93 (1.56, 2.32)	3.97 (3.50, 4.44)	2.44 (2.02, 2.86)
80+	3.00 (2.54, 3.46)	2.57 (1.97, 3.18)	1.81 (1.35, 2.27)	3.52 (2.93, 4.11)	2.43 (1.91, 2.95)
Hispanic					
Yes	3.32 (3.13, 3.52)	3.64 (3.39, 3.89)^a^	1.84 (1.64, 2.63)	4.26 (4.02, 4.51)^a^	2.82 (2.60, 3.54)^a^
No	3.13 (2.97, 3.29)	3.11 (2.90, 3.32)^b^	1.64 (1.48, 1.82)	3.91 (3.70, 4.11)^b^	2.39 (2.21, 2.57)^b^
Race					
White	3.24 (3.09, 3.39)	3.30 (3.11, 3.49)	1.71 (1.57, 1.86)	3.96 (3.77, 4.15)	2.54 (2.36, 2.78)
Black	3.31 (2.85, 3.77)	3.48 (2.87, 4.28)	1.78 (1.32, 2.24)	4.10 (3.50, 4.69)	2.50 (1.97, 3.52)
Asian	4.00 (2.52, 5.48)	2.50 (0.54, 4.46)	2.83 (1.34, 4.33)	4.50 (2.57, 6.43)	3.25 (1.55, 4.95)
Native Hawaiian or Other Pacific Islander	2.00 (0.52, 3.48)	2.00 (0.04, 3.96)	2.00 (0.51, 3.49)	5.00 (3.07, 6.93)	3.50 (1.80, 5.20)
American Indian or Alaska Native	2.00 (0.95, 3.15)	3.00 (1.61, 4.39)	2.50 (1.44, 3.56)	4.50 (3.14, 5.86)	2.69 (1.49, 3.89)
Mixed	3.18 (2.82, 3.55)	3.18 (2.70, 3.66)	1.61 (1.24, 1.97)	4.27 (3.80, 4.75)	2.56 (2.14, 2.98)
Other	3.07 (2.62, 3.52)	3.86 (3.27, 4.45)	1.65 (1.20, 2.11)	4.32 (3.73, 4.96)	2.72 (2.20, 3.23)
Language					
English only^a^	3.10 (2.94, 3.27)	3.16 (2.95, 3.38)^c^	1.60 (1.44, 1.77)	4.06 (3.85, 4.27)	2.42 (2.23, 2.68)^d,e^
Spanish only^b^	3.65 (3.18, 4.12)	3.50 (2.89, 4.11)	1.55 (1.08, 2.32)	3.95 (3.34, 4.56)	2.21 (1.69, 2.73)^d,e^
Both, more English than Spanish^c^	3.25 (2.85, 3.65)	3.96 (3.45,4.48)^a,f^	1.70 (1.30, 2.18)	4.07 (3.56, 4.58)	2.54 (2.09, 2.98)^d^
Both, equal amounts^d^	3.10 (2.63, 3.57)	3.65 (3.04, 4.26)	2.13 (1.66, 2.66)	4.25 (3.64, 4.86)	3.48 (2.95, 4.29)^a,b,c,f^
Both, more Spanish than English^e^	3.43 (3.07, 3.79)	3.59 (3.11, 4.96)	2.12 (1.76, 2.48)	4.38 (3.92, 4.85)	2.89 (2.49, 3.29)^a,b^
Other^f^	3.25 (2.84, 3.66)	2.92 (2.39, 3.46)^c^	1.78 (1.37, 2.19)	3.46 (2.93, 3.99)	2.62 (2.15, 3.77)^d^
Employment status					
Full time	3.47 (2.92, 4.31)	3.07 (2.35, 3.78)	1.62 (1.07, 2.17)	3.47 (2.77, 4.17)	2.48 (1.86, 3.16)
Part time	3.17 (2.78, 3.56)	2.93 (2.42, 3.44)	1.67 (1.27, 2.36)	4.31 (3.81, 4.81)	2.90 (2.45, 3.34)
Unemployed	3.22 (2.95, 3.49)	3.53 (3.17, 3.89)	1.74 (1.46, 2.42)	4.29 (3.93, 4.64)	2.61 (2.30, 2.92)
Disabled	3.38 (3.15, 3.66)	3.52 (3.23, 3.81)	1.76 (1.54, 1.99)	3.94 (3.66, 4.23)	2.58 (2.32, 2.83)
Retired	2.98 (2.72, 3.24)	3.14 (2.80, 3.48)	1.77 (1.51, 2.14)	3.98 (3.65, 4.32)	2.37 (2.07, 2.66)
Student/Homemaker	3.06 (2.69, 3.43)	3.25 (2.76, 3.74)	1.56 (1.19, 1.94)	4.09 (3.61, 4.57)	2.58 (2.15, 3.52)
Education					
< First grade-9th grade^g^	3.40 (3.16, 3.64)	3.49 (3.18, 3.86)^i^	1.87 (1.62, 2.17)	4.22 (3.91, 4.53)	2.54 (2.26, 2.81)
Grades 10-12^h^	3.16 (2.98, 3.33)	3.51 (3.29, 3.73)^i^	1.76 (1.59, 1.93)	4.01 (3.79, 4.23)	2.55 (2.35, 2.74)
Vocational/Tech/some college^i^	3.14 (2.85, 3.44)	2.60 (2.22,2.97)^g,h^	1.43 (1.14, 1.72)	3.88 (3.51, 4.26)	2.52 (2.19, 2.86)
Bachelor’s Degree/Post-graduate^j^	2.75 (2.08, 3.42)	3.00 (2.15, 3.85)	1.50 (0.84, 2.16)	4.20 (3.33, 5.86)	3.23 (2.47, 3.98)
Income (yearly)					
< $6000^j^	3.41 (2.91, 3.91)	3.71 (3.02, 4.39)	2.10 (1.58, 2.62)	4.65 (4.00, 5.29)	3.13 (2.57, 3.69)^l,m^
$,6000-$11,9999^k^	3.33 (3.12, 3.54)	3.30 (3.01, 3.59)	1.91 (1.69, 2.13)	4.22 (3.94, 4.59)	2.69 (2.45, 2.93)^l^
$12,000-$17,999^l^	3.30 (3.06, 3.54)	3.32 (3.00, 3.65)	1.62 (1.37, 1.87)	3.81 (3.50, 4.12)	2.31 (2.04, 2.58)^j,k^
$18,000 + ^m^	3.03 (2.76, 3.31)	3.45 (3.08, 3.82)	1.59 (1.30, 1.87)	4.14 (3.79, 4.49)	2.45 (2.14, 2.75)^j^

^a–f^denote significant differences between language groups p < 0.05

^g–i^denote significant differences between education levels *p* < 0.01

^j–m^denote significant differences by income level *p* < 0.05

Table 4 presents the regression models examining associations of neighborhood-, friend-, and family- norms and social support for F&V with the outcomes, fruit, vegetable, and F&V intake, with adjustments for housing site, gender, age, race, ethnicity, and education. The family norm for F&V was associated with a 0.18 cup increase in fruit (95% CI = 0.06, 0.31). Neighborhood social support for F&V was associated with a 0.22 cup increase in vegetables (95% CI = 0.04, 0.39). The family norm for F&V was associated with a 0.14 cup increase in vegetables (95% CI = 0.0, 0.28) and friend and family social support for F&V was associated with a 0.30 cup increase in vegetables (95% CI = 0.15, 0.60). Neighborhood social support for F&V was associated with a 0.31 cup increase in F&V (95% CI = 0.05, 0.57). The family norm for F&V was associated with a 0.32 cup increase in F&V (95% CI = 0.13, 0.52) and friend and family social support for F&V was associated with a 0.42 cup increase in F&V (95% CI = 0.19, 0.64).

**Table 4:** Regression Models Examining Norms and Social Support on Fruit, Vegetable, and Combined Fruit and Vegetable (F&V) Intake; Unstandardized beta (95% confidence interval)

Neighborhood questions	Fruit	*P*-value	Vegetables	*P*-value	F&V Combined	*P*-value
	b (95% CI)		b (95% CI)		b (95% CI)	
Neighborhood Injunctive Norm for F&V	0.10 (−0.06, 0.26)	0.24	0.02 (−0.16, 0.20)	0.84	0.12 (0.15, 0.38)	0.39
Neighborhood Descriptive Norm for F&V	0.06 (−0.07, 0.18)	0.38	−0.02 (− 0.17, 0.12)	0.75	0.03 (− 0.18, 0.24)	0.79
Neighborhood Social Support for F&V	0.10 (− 0.06, 0.26)	0.23	0.22 (0.04, 0.39)	0.02	0.31 (0.05, 0.57)	0.02
Family Norm for F&V	0.18 (0.06, 0.31)	< 0.01	0.14 (0.00, 0.28)	0.04	0.32 (0.13, 0.52)	0.001
Family and Friend Social Support for F&V	0.11 (− 0.03, 0.25)	0.12	0.30 (0.15, 0.60)	< 0.001	0.42 (0.19, 0.64)	< 0.001

*Note*. *B* unstandardized beta, *CI* 95% confidence interval. All models are adjusted for site, gender, age, race, ethnicity, education, and employment status

## Discussion

This study examined associations between perceived neighborhood-, friend-, and family- norms and social support for F&V with F&V intake among residents in low-income subsidized housing communities. Findings from the current study provide support for the Reserve Capacity Model which postulates that a reserve of adaptive and social resilience resources like neighborhood, family, and peer social support may help low-SEP individuals overcome some of the adversities of disadvantaged economic environments.

Our study findings add to a growing body of literature describing the associations between perceived neighborhood social environments and diet-related behaviors [21, 41, 42]. To our knowledge, our study is the first to provide empirical evidence that perception of neighborhood social support for F&V purchase and consumption is associated with self-reported F&V intake. These findings are particularly noteworthy because neighborhood social support remained significant even after accounting for peer- and family- norms and social support. Neighborhood social support was associated with an almost one-third cup increased intake of F&V combined, which accounts for a sizable proportion of the recommended daily servings of F&V [1]. The null findings for neighborhood descriptive and injunctive norms for F&V with F&V intake may result from the types of questions asked and thus warrants further exploration. Recent research suggests that descriptive norms may be more important for adopting specific dietary behaviors [22, 43]. As such, future studies should examine the potential role of perceived neighborhood descriptive norms and neighborhood social support for both healthy and unhealthy dietary behaviors (e.g., F&V and sugar sweetened beverages) in other populations and within the context of dietary interventions [44–46].

The findings from the current study are consistent with studies showing that perceived friend and family social norms and support influence adolescents’ and adults’ F&V intake [36, 47–49]. Our findings align with those of Ball et al., who report that friend and family norms for healthy eating are associated with F&V intake [36]. However, in the results from the work of Ball et al., social norms are not significantly associated with F&V after controlling for family and friend social support for healthy eating. These finding contrast with findings from the current study which indicate that both friend and family social norms and social support for F&V are independently associated with F&V intake. Furthermore, similar to previous studies, our results suggest that friend and family social support are associated with F&V intake [31, 40]. However, our finding of a protective effect of social support on diet contrasts with the finding of Tamers and colleagues [37]. This discrepancy may be due to differences in measurement of social support as Tamers and colleagues used a general measure of instrumental and emotional social support [37] while we examined specific types of social support for health behaviors, which may be a better predictor of dietary behaviors. Future studies should examine the relationship between dietary intake and different types of social support.

While the present study establishes significant associations between perceived neighborhood social support for F&V and F&V intake, it does have some limitations. First, since the ancillary study only had available data from four of the 4 intervention sites, we were unable to account for clustering of individuals within housing sites. Instead, we controlled for housing site as an independent level variable. Second, because of the limited number of housing sites, we were unable to assign a norm or social support value at the neighborhood level. Because of this limitation, we examined residents’ perceptions of neighborhood norms and social support at the individual level. Third, the Live Well intervention study only explored F&V-related factors so we were unable to examine other types of dietary norms related to unhealthy dietary patterns (e.g., added sugars, saturated fats, sodium) that are known to increase disease risk. Fourth, this study relied on self-reported F&V intake using a validated questionnaire; however, using biomarkers of F&V intake such as serum carotenoids or 24-h dietary recall data would help to minimize bias due to measurement error and in turn strengthen the current study findings. Finally, the research findings are cross-sectional, so we cannot infer causation. Future studies should examine longitudinal associations to determine causality.

## Conclusions

This study provides quantitative evidence that neighborhood social support is associated with F&V intake independent of peer- and family- norms and social support. The findings suggest that neighborhood social environments characterized by social support for purchase and consumption of F&V may be associated with increased F&V intake despite the challenges associated with low neighborhood SEP. Our findings offer a potential explanation for mixed findings regarding objective neighborhood food environments and diet quality. Neighborhood social support for healthy eating may need to be considered when developing neighborhood interventions and/or policies to reduce neighborhood disparities in F&V access, availability, and diet-related behaviors.

## Abbreviations

(F&V: Fruit and vegetable
NCI: National Cancer Institute
PCA: Principal Components Analysis
SEP: Socioeconomic Position
U.S.: United States
